# Perturbations of Glutathione and Sphingosine Metabolites in Port Wine Birthmark Patient-Derived Induced Pluripotent Stem Cells

**DOI:** 10.3390/metabo13090983

**Published:** 2023-08-31

**Authors:** Vi Nguyen, Jacob Kravitz, Chao Gao, Marcelo L. Hochman, Dehao Meng, Dongbao Chen, Yunguan Wang, Anil G. Jegga, J Stuart Nelson, Wenbin Tan

**Affiliations:** 1 Department of Cell Biology and Anatomy, School of Medicine, University of South Carolina, Columbia, SC 29209, USA; vi.nguyen@uscmed.sc.edu (V.N.); kravitzj@email.sc.edu (J.K.); chao.gao@uscmed.sc.edu (C.G.); 2 The Facial Surgery Center and the Hemangioma & Malformation Treatment Center, Charleston, SC 29425, USA; drhochman@facialsurgerycenter.com; 3 Department of Otolaryngology—Head and Neck Surgery, Medical University of South Carolina, Charleston, SC 29425, USA; 4 Applied Physics Program, California State University San Marcos, San Marcos, CA 92096, USA; meng004@csusm.edu; 5 Department of Obstetrics and Gynecology, University of California, Irvine, CA 92617, USA; dongbaoc@uci.edu; 6 Department of Pediatrics, University of Cincinnati College of Medicine, Cincinnati, OH 45229, USA; yunguan.wang@cchmc.org (Y.W.); anil.jegga@cchmc.org (A.G.J.); 7 Division of Gastroenterology, Cincinnati Children Hospital Medical Center, Cincinnati, OH 45229, USA; 8 Division of Human Genetics, Cincinnati Children Hospital Medical Center, Cincinnati, OH 45229, USA; 9 Division of Biomedical Informatics, Cincinnati Children Hospital Medical Center, Cincinnati, OH 45229, USA; 10 Departments of Surgery and Biomedical Engineering, Beckman Laser Institute and Medical Clinic, University of California, Irvine, CA 92617, USA; jsnelson@uci.edu; 11 Department of Biomedical Engineering, College of Engineering and Computing, University of South Carolina, Columbia, SC 29208, USA

**Keywords:** Port Wine Birthmark, induced pluripotent stem cells, metabolome, glutathione, sphingolipid, sphingosine, hypoxia

## Abstract

Port Wine Birthmarks (PWBs) are a congenital vascular malformation on the skin, occurring in 1–3 per 1000 live births. We have recently generated PWB-derived induced pluripotent stem cells (iPSCs) as clinically relevant disease models. The metabolites associated with the pathological phenotypes of PWB-derived iPSCs are unknown, and so we aim to explore them in this study. Metabolites were separated by ultra-performance liquid chromatography and screened with electrospray ionization mass spectrometry. Orthogonal partial least-squares discriminant, multivariate, and univariate analyses were used to identify differential metabolites (DMs). KEGG analysis was used to determine the enrichment of metabolic pathways. A total of 339 metabolites was identified. There were 22 DMs, among which nine were downregulated—including sphingosine—and 13 were upregulated, including glutathione in PWB iPSCs, as compared to controls. Pathway enrichment analysis confirmed the upregulation of glutathione and the downregulation of sphingolipid metabolism in PWB-derived iPSCs as compared to normal ones. We next examined the expression patterns of the key molecules associated with glutathione metabolism in PWB lesions. We found that hypoxia-inducible factor 1α (HIF1α), glutathione S-transferase Pi 1 (GSTP1), γ-glutamyl transferase 7 (GGT7), and glutamate cysteine ligase modulatory subunit (GCLM) were upregulated in PWB vasculatures as compared to blood vessels in normal skin. Other significantly affected metabolic pathways in PWB iPSCs included pentose and glucuronate interconversions; amino sugar and nucleotide sugars; alanine, aspartate, and glutamate; arginine, purine, D-glutamine, and D-glutamate; arachidonic acid, glyoxylate, and dicarboxylate; nitrogen, aminoacyl-tRNA biosynthesis, pyrimidine, galactose, ascorbate, and aldarate; and starch and sucrose. Our data demonstrated that there were perturbations in sphingolipid and cellular redox homeostasis in PWB vasculatures, which could facilitate cell survival and pathological progression. Our data implied that the upregulation of glutathione could contribute to laser-resistant phenotypes in some PWB vasculatures.

## 1. Introduction

Congenital capillary malformations, also known as port-wine birthmarks or stains (PWB or PWS), are the most common types of congenital vascular malformations (CVMs). PWBs result from developmental defects in the skin vasculature, with an estimated prevalence of 1–3 per 1000 live births [1,2,3,4]. The skin lesions can be isolated or in association with other CVMs in children [5,6]. At birth, PWBs appear as flat red macules; lesions progressively darken to purple during the growth of afflicted children. By middle age, lesions in many patients show the development of vascular nodules that are susceptible to spontaneous bleeding or hemorrhage [1,2,3,4]. Moreover, the quality of life of PWB children is greatly affected during their development and growth due to the condition’s devastating lifelong psychological and social impacts [1,7].

PWB lesions typically exhibit a progressive dilatation of the dermal capillaries, the proliferation of endothelial cells (ECs) and smooth muscle cells (SMCs), the replication of basement membranes, the disruption of vascular barriers, and the expression of endothelial progenitor cell biomarkers [8,9,10,11]. Pulsed dye lasers (PDLs) are the treatment of choice for PWB. Unfortunately, less than 10% of patients achieve the complete removal of PWB after multiple PDL treatments, and approximately 20% of lesions show little or no response to laser exposure [12,13]. A recent review showed that there has been little improvement in clinical outcomes using laser-based modalities for PWB treatment over the past three decades [14]. 

The absence of clinically relevant cell and animal models has been a long-term obstacle to the understanding of the pathogenesis of and the development of therapeutics for PWB. In an effort to overcome this barrier, we have recently generated PWB patient-derived induced pluripotent stem cells (iPSCs) by introducing “Yamanaka factors” (Oct3/4, Sox2, Klf4, c-Myc) [15] into lesional dermal fibroblasts. These disease iPSCs can be differentiated into ECs that recapulate many of the pathological phentoypes of the PWB vasculature, including the formation of an enlarged vasculature in vitro and in vivo [16]. However, the metabolites reflecting the pathological phenotypes of PWB-derived iPSCs are unknown. The identification of metabolic signatures from PWB-derived iPSCs will provide us with new insights into the crucial signalosomes that lead to the development of a lesional vasculature. 

## 2. Materials and Methods

### 2.1. Tissue Preparation

This study (#1853132) was approved by the Institutional Review Board at Prisma Health Midlands. For iPSC generation, surgically excised nodular PWB lesions from two patients (both male, Fitzpatrick II skin type, 45–60 years old) and one age and gender-matched de-identified surgically discarded piece of normal skin tissue were collected. For the immunohistochemistry (IHC) studies, de-identified surgically excised hypertrophic and nodular PWB lesions (*n* = 4) and de-identified surgically discarded normal skin tissue (*n* = 5) were collected through our previous studies [8,11,17,18]. 

### 2.2. PWB iPSC Culture and Sample Preparation

The generation of the PWB and control iPSC lines from skin biopsies are detailed in our recent study [16]; these cell lines were maintained and propagated under feeder-free conditions using an Essential 8 Medium on a geltrex-coated plate (ThermoFisher, Waltham, MA, USA). The following iPSC lines were used in this study: PWB_4221_3, PWB_4221_6, PWB_3921_9, PWB_3921_16, control_52521_8, and control_52521_9 [16]. There were experimental duplications for PWB_4221_3, PWB_3921_9, and control_52521_8, resulting in a total of 9 samples. The iPSCs (~10^6^ cells) were dissociated using StemPro Accutase (ThermoFisher, Waltham, MA, USA); cell pellets were collected and stored at −80 °C until processing. Samples were thawed with the addition of 300 µL of 80% methanol. Samples were vortexed for 60 s, sonicated for 30 min at 4 °C, and incubated at −20 °C for one hour. The samples were then subjected to centrifugation at 12,000 rpm at 4 °C for 15 min. The supernatant (200 µL) was mixed with 5 µL of L-o-Chlorophenylalanine (0.14 mg/mL) for liquid chromatography–mass spectrometry (LC–MS) analysis. 

### 2.3. Metabolomics by LC–MS

For metabolomics analysis by LC–MS, separation was carried out using the Vanquish Flex Ultra-performance liquid chromatograph (UPLC) along with the Q Exactive MS (ThermoFisher, Waltham, MA, USA), and it was screened with electrospray ionization MS (ESI-MS). The LC system was comprised of the ACQUITY UPLC HSS T3 (100 × 2.1 mm × 1.8 μm) with the UPLC. The mobile phase was composed of 0.05% formic acid water and acetonitrile with gradient elution (0-1 min, 5% acetonitrile; 1–12 min, 5–95% acetonitrile; 12–13.5 min, 95% acetonitrile; 13.5–13.6 min, 95–5% acetonitrile; 13.6–16 min, 5% acetonitrile). The flow rate of the mobile phase was 0.3 mL/min. The column temperature was maintained at 40 °C, and the sample manager temperature was set at 4 °C. MS in ESI+ and ESI− mode were listed as follows: (1) ESI+: Heater Temp 300 °C; Sheath Gas Flow rate, 45 arb; Aux Gas Flow Rate, 15 arb; Sweep Gas Flow Rate, 1 arb; spray voltage, 3.0 KV; Capillary Temp, 350 °C; S-Lens RF Level, 30%; and (2) ESI−: Heater Temp 300 °C, Sheath Gas Flow rate, 45 arb; Aux Gas Flow Rate, 15 arb; Sweep Gas Flow Rate, 1 arb; spray voltage, 3.2 KV; Capillary Temp, 350 °C; S-Lens RF Level, 60%.

### 2.4. Immunohistochemistry (IHC)

IF microscopy was performed to access biomarker expression in iPSCs growing on Geltrex-coated coverslips. The cells were fixed using 4% buffered paraformaldehyde for 10 minutes, blocked using 5% normal donkey serum for 1 h at room temperature, and followed by overnight incubation with the following primary antibodies at 4 °C (1:100 dilution): anti-Tra1-60 (Abcam, Cambridge, UK, #ab16288), anti-Nanog (Abcam, #ab21624), anti-Sox2 (Abcam, #ab79351), and anti-Oct4 (Abcam, #ab19857). Alexa488 or Alexa555 fluorescent-conjugated anti-rabbit or mouse secondary antibodies (ThermoFisher, 1:200 dilution) were incubated with sections for 1 h at room temperature after the primary antibodies’ reaction. Negative controls were performed without primary antibodies. Images were acquired using a Leica Stellaris 5 DMS CS confocal microscope.

For IHC, skin biopsies were fixed in 4% buffered paraformaldehyde overnight and embedded in paraffin. The paraffin sections (6 µm thickness) were deparaffinized in 100% xylene for 10 min for three times. The sections then went through graded alcohols from 100%, 80%, to 70% and rehydrated in distilled water. Antigen retrieval was performed in 10 mM sodium citrate buffer (pH 6.0) at 97 °C for 2 h. Sections were treated with 3% hydrogen peroxide for 30 min and blocked using 5% normal horse serum for 1 h at room temperature. Sections were then incubated in a humidified chamber overnight at 4 °C with the following primary antibodies: anti-hypoxia-inducible factor 1α (HIF1α) (Santa Cruz Biotech., Dallas, TX, USA; #SC-10790; 1:100 dilution); anti-glutathione S-transferase Pi 1 (GSTP1; Proteintech, #15902-1-AP; 1:1000 dilution), anti-γ-glutamyl transferase 7 (GGT7) (Proteintech, Rosemont, IL, USA; #24674-1-AP; 1:1000 dilution), and anti- glutamate cysteine ligase modulatory subunit (GCLM; Proteintech, #14241-1-AP; 1:1000 dilution). Biotinylated anti-mouse or -rabbit secondary antibodies (Vector laboratories, Newark, CA, USA) (1:250 dilution) were incubated with sections for 2 hrs at room temperature after the primary antibody reaction. An indirect biotin avidin diaminobenzidine (DAB) system (Vector laboratories, Newark, CA, USA) was used for detection based on the manufacturer’s manual. IHC scores were developed as previously reported [17] and tailored for individual blood vessels using the following two factors: (1) an intensity factor ranging from 0 to 5, which was the average intensity of all the ECs in one blood vessel; Each EC was graded using the following categories: no staining, 0; very weak staining, 1; mild staining, 2; intermediate staining, 3; strong staining, 4; and very strong staining, 5; and, (2) a percentage factor also ranging from 0 to 5, which was equal to the multiplication of the percentage (%) of immunoreactive-positive ECs in one blood vessel by a factor of 5. For each blood vessel, an antibody immunoreactivity score was estimated by multiplying the intensity and percentage factors, ranging from 0 to 30. 

### 2.5. Statistical Analysis

The raw data were acquired and aligned using the Compound Discoverer (3.0, Thermo) based on the *m*/*z* value and the retention time of the ion signals. Ions from both ESI− and ESI+ were merged and imported into the SIMCA-P program (version 14.1) for multivariate analysis. Principal Component Analysis (PCA) was used for data visualization and outlier identification. Supervised regression modeling was then performed on the data set using Partial Least Squares Discriminant Analysis (PLS-DA) or Orthogonal Partial Least Squares Discriminant Analysis (OPLS-DA) to identify the potential dysregulated metabolites. The metabolites were filtered and confirmed by combining the results of the Variable Importance in Projection (VIP) values (VIP > 1.5) and t-test values (*p* < 0.05). The quality of the fitting model was explained by the R2 and Q2 values. R2 displayed the variance explained by the model and indicated the quality of the fit. Q2 displayed the variance in the data, indicating the model predictability. The final metabolic signatures were confirmed using the data on the accurate masses and MS/MS fragments with the criteria of a VIP score > 1.5, absolute log2(fold change) > 0.5, and *p* value < 0.05. Hierarchical cluster analysis (HCA) was performed using the complete linkage algorithm of the program Cluster 3.0 and the results are visualized using Pheatmap 1.0.12 (Raivo Kolde).

## 3. Results

**Figure 1:**
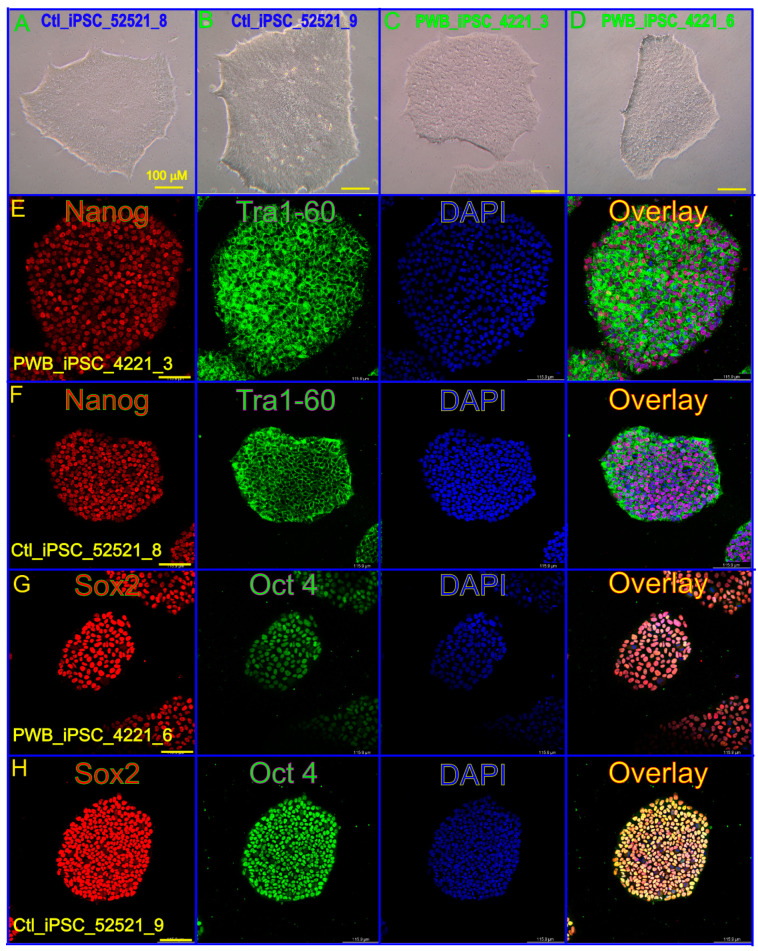
Characterization of normal skin and PWB-derived iPSCs. (**A**–**D**) Normal and PWB disease iPSC colonies under a bright field. (**E**–**H**) Stem cell biomarkers Nanog (**E**), Tra1-60 (**F**), Sox 2 (**G**), and Oct4 (**H**) were used to verify the control and PWB iPSC cells. DAPI: nuclei staining. Scale bar: 100 µm.

**Figure 2:**
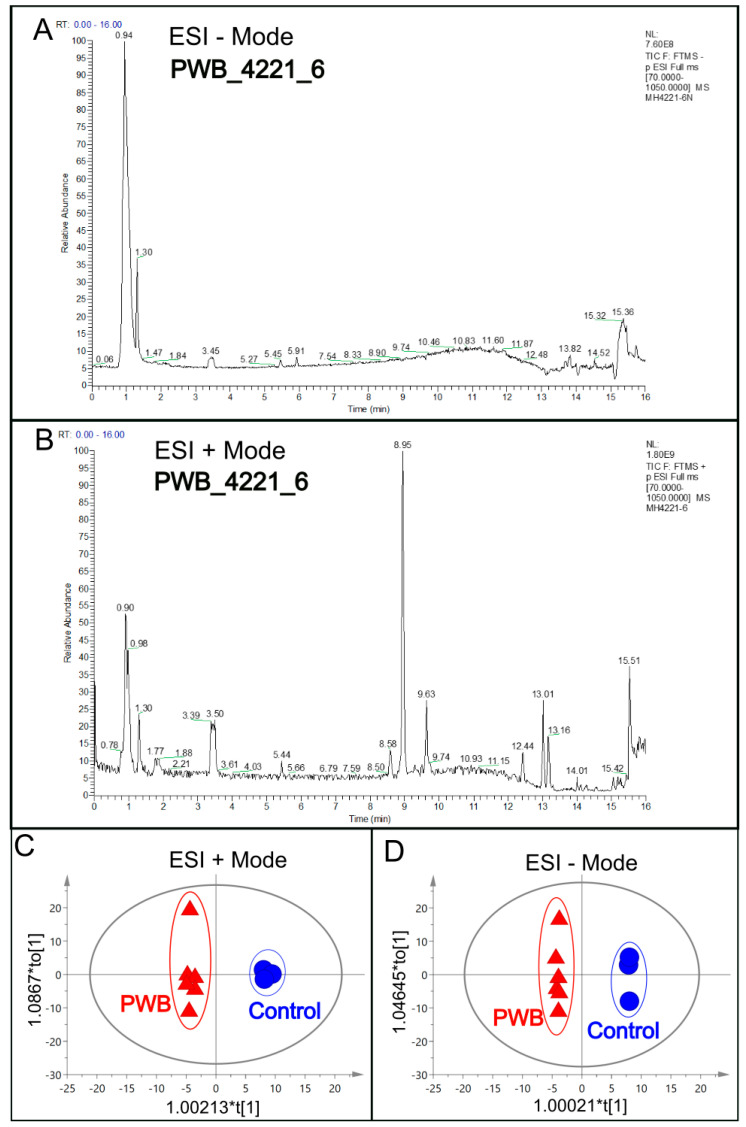
An example of a total ion chromatogram (TIC), representing the summed intensity with all detected mass spectral peaks associated with metabolites. (**A**), TIC from the PWB_4221_6 iPSC line in a negative ion mode electrospray ionization (ESI−); (**B**), TIC from the same iPSC line in positive ion mode electrospray ionization (ESI+); (**C**), OPLS-DA model showing scattering scores and cluster tendencies among all samples in ESI + mode; (**D**), OPLS-DA model showing scattering scores and cluster tendencies among all samples in ESI − mode. Digits on the X or Y axis are eigenvalues of the regression coefficient for the predictive principal component (X) or the orthogonal component (Y), respectively.

The expression of stem cell biomarkers such as Nanog, Tra1-60, Sox 2, and Oct4 in both the control and PWB iPSC lines was verified using IF (Figure 1). The cellular metabolites were extracted from these iPSC lines for LC–MS analysis. The total ion chromatograms (TIC) showed the summed-up intensities of all the mass spectral peaks associated with metabolites in the samples (Figure 2A,B, Figure S1 and S2). The data were normalized after alignment. For each mode (ESI− or ESI+), five internal quality control samples were investigated (Supplementary Figures S1A and S2A). PCA showed that the quality control samples were highly clustered, but no clear grouping trend was observed between the PWB and control groups (Supplementary Figure S3A,B). To eliminate any non-specific effects and to confirm the identified dysregulated metabolites, PLS-DA or OPLS-DA was used to compare metabolic changes between the two groups, respectively. Both analyses showed a clear separation of the PWB and control groups (Figure 2C,D, Figure S4 and S5). A total of 339 metabolites were detected amongst the samples. There were 156 metabolites detected in the ESI− mode, 201 in the ESI+ mode, and 18 overlapping metabolites detected in both modes. 

**Figure 3:**
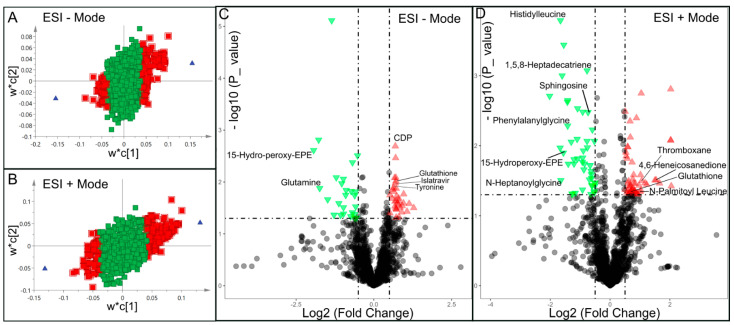
Discovery of differential metabolites (DMs) by multivariate analysis. A and B, distribution of significant metabolites detected in ESI− (**A**) and ESI+ (**B**) modes using a PLS-DA model resulting in coefficients for the variables in a w*c loading plot. w, PLS-weights for the X-variables; c, PLS-weights for the Y-variables; Red box, metabolites with VIP > 1.5; green box: metabolites with VIP < 1.5; Blue triangle is symbolized by two group dots and X-variables located near a group dot are positively associated with that group; C and D, volcano plots showing clusters of DMs detected in ESI− (**C**) and ESI+ (**D**) modes. Green triangle, significantly downregulated metabolites; red triangle: significantly upregulated metabolites; black circle: insignificant metabolites; vertical dash lines: log_2_ (fold change) = ± 0.5; horizontal dash line: *p* value = 0.05.

**Figure 4:**
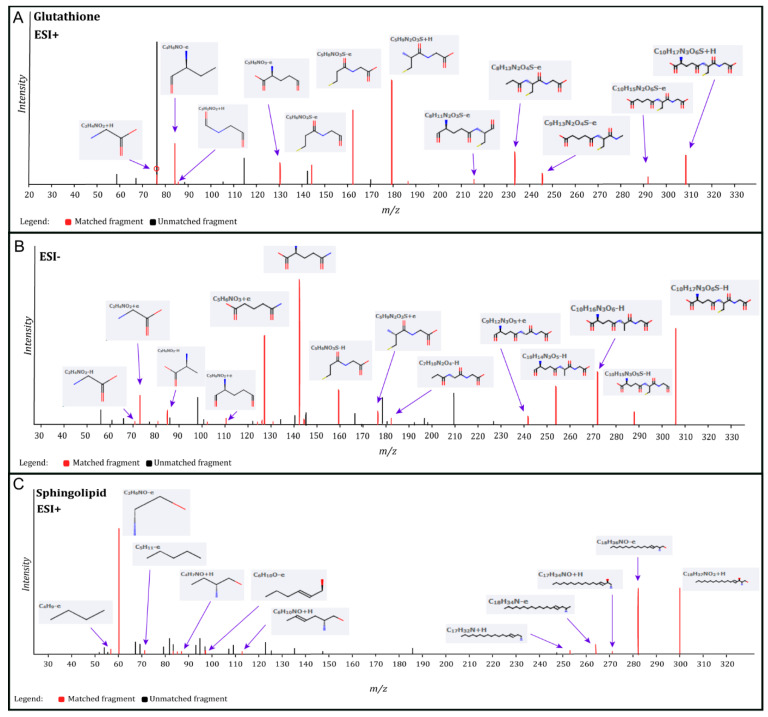
Identification of glutathione and sphingosine metabolites. (**A**), identified matched glutathione MS fragments in ESI+ mode; (**B**), identified matched glutathione MS fragments in ESI− mode; (**C**), identified matched sphingosine MS fragments in ESI+ mode. The MS chromatograms of glutathione and sphingosine fragments from each sample were extracted and aligned into one chromatogram.

**Figure 5:**
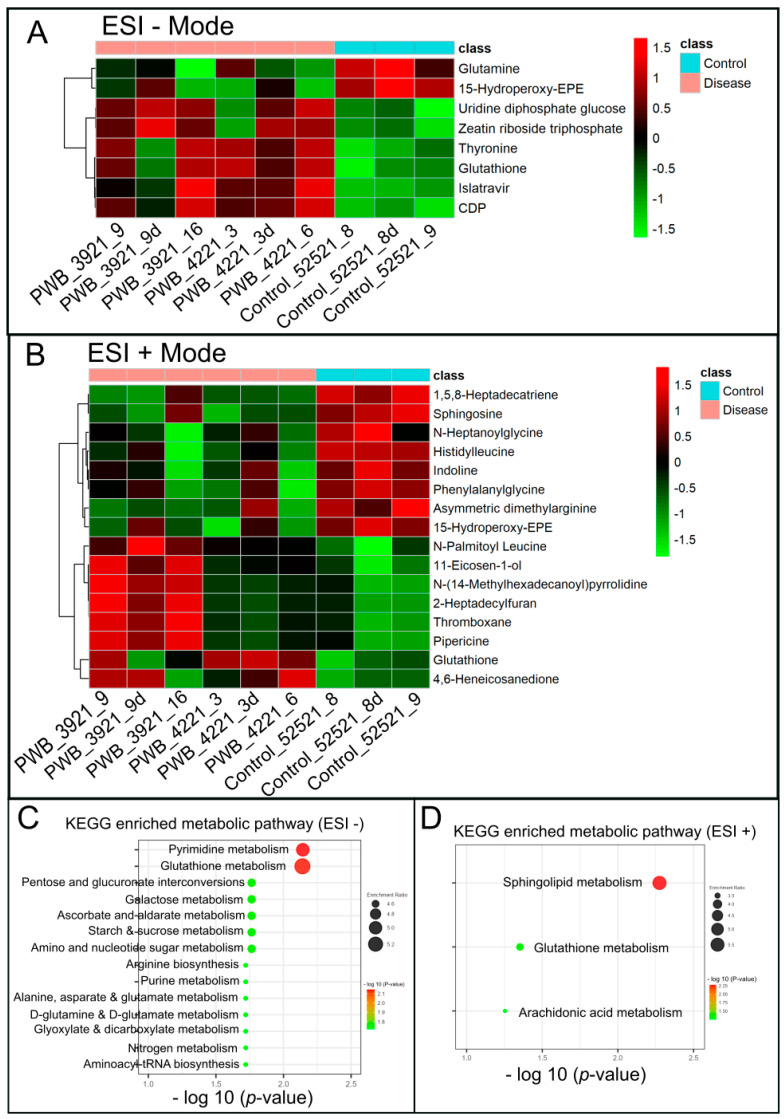
Hierarchical cluster analysis (HCA) and KEGG pathway enrichment of metabolome data. (**A**,**B**), the heatmap of significantly DMs identified in ESI− (**A**) and ESI+ mode (**B**) in PWB iPSCs as compared to the control ones; C and D, enriched KEGG pathways related to the perturbed metabolic networks involving DMs identified in ESI − (**C**) and ESI + mode (**D**); the enriched metabolic pathways are indicated by color (−log_10_ (*p* value)) and size (black ball, enrichment ratio). PWB_3921_9d, PWB_4221_3d, and Control_52521_8d were experimental duplications of PWB_3921_9, PWB_4221_3, and Control_52521_8, respectively.

Significantly changed metabolites between the two groups were called out based on their VIP scores (VIP > 1.5; Supplementary Figure S6). The metabolic signatures were filtered and collected by combining the results of the VIP scores (VIP > 1.5) and the *t*-test values (*p* < 0.05; Figure 3A,B). The significantly differentiated metabolites (DMs; absolute log2(fold change) > 0.5, and *p* value < 0.05) were determined using univariate analysis (Figure 3C,D). There was an upregulation of glutathione and a downregulation of sphingosine in the PWB iPSCs as compared to the control ones. The representative mass spectra for glutathione and sphingosine are shown in Figure 4 and Supplementary Table S1. A total of 22 DMs were identified in the final list (8 in ESI− and 16 in ESI+, with two overlapped ones; Table 1, Figure 5A,B). Next, the metabolic network was revealed using KEGG enrichment analysis (Figure 5C,D). Significantly affected metabolic pathways included sphingolipid, glutathione, pentose and glucuronate interconversions; amino sugars and nucleotide sugars; alanine, aspartate, and glutamate; arginine, purine, D-glutamine, and D-glutamate; arachidonic acid, glyoxylate, and dicarboxylate; nitrogen, aminoacyl-tRNA biosynthesis, pyrimidine, galactose, ascorbate, and aldarate; and starch and sucrose. 

**Figure 6:**
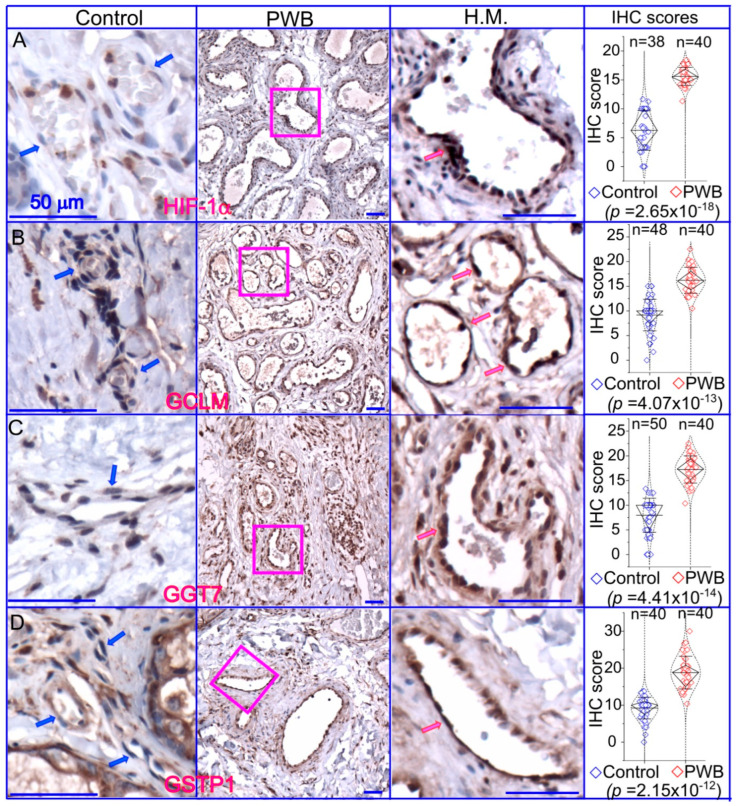
Expressions of key molecules associated with glutathione metabolism in PWB lesions. The IHC assays using antibodies against HIF−1α (**A**), GCLM (**B**), GGT7 (**C**), and GSTP1 (**D**) show the immunoreactive blood vessels in control skin or PWB lesions. H.M., a higher magnification from the pink boxed area in the left panel showing immunoreactive positive PWB blood vessels for the corresponding antibodies. Scale bar: 50 µm. n, number of blood vessels from PWB (4 subjects) or normal ones (5 subjects). Whiskers: mean ± S.D.; Diamond boxes: IQR; Dotted curves: data distribution. Paired *t*-test was used for comparing arbitary IHC scores. Blue arrows: control dermal capillaries; red arrows: PWB blood vessels.

Last, we focused on the glutathione pathway in PWB lesions by examining the expression patterns of HIF1α and several key enzymes related to glutathione metabolism, including GSTP1, GGT7, and GCLM. The ECs in normal skin showed mild or moderate immunoreactive signals for the antibodies examined (Figure 6), which were also consistent with the IHC data on human skin from the Human Protein Atlas (https://www.proteinatlas.org/; accessed on 7 June 2023). In PWB lesions, these antibodies showed moderate to strong immunoreactive signals on ECs (Figure 6 and Figure S7). The immunoreactive scores of these antibodies were significantly higher in the PWB vasculature than in normal dermal blood vessels (Figure 6). 

## 4. Discussion

In this study, we have shown a downregulation of sphingosine and an upregulation of glutathione in PWB iPSCs through a non-targeted metabolomics study, which is the first of its kind. The PWB vasculature exhibited elevated HIF−1α levels, demonstrating persistent and mild hypoxia as a driving force increasing glutathione. The dysregulation of crucial enzymes involved in glutathione metabolism in PWB lesions further supports our metabolic data. Our study provides the first link between metabolic signatures such as sphingosine and glutathione and the pathological progression of PWB, opening new avenues of investigations in the field. 

Our results imply a potential association between sphingolipids and the pathogenesis of PWB. Ceramide, sphingosine, and their derived sphingolipids are active lipids that execute diverse cellular signaling [19,20]. Ceramide is produced by the breakdown of membrane sphingomyelin by sphingomyelinase or de novo synthesis. In contrast, sphingosine is generated through the de-acylation of ceramide by ceramidase. Sphingosine can further generate sphingosine-1-phosphate (S1P) by sphingosine kinases. Sphingosine and sphingolipids have been shown to have multiple biological functions, including pro-apoptosis, cell cycle arrest, cytoskeleton regulation, endocytosis, autophagic process, and antimicrobial effects [21,22,23,24,25,26]. Sphingosine is considered an endogenous inhibitor of protein kinase C [23,27,28]; it also induces apoptosis via the activation of PKA and the inhibition of MAPK [19,20]. Our previous studies have shown an activation of PKC, MAPK, and PI3K pathways in the PWB vasculature [4,17,29]. These data suggest two possible mechanisms by which sphingosine may contribute to the pathological development of PWB: First, downregulated sphingosine in PWB lesions may cause lower inhibition of PKC and MAPK, leading to cell proliferation. Second, a low level of sphingosine may attenuate PDL-induced apoptosis due to its pro-apoptotic function; however, the detailed mechanisms are yet to be determined. 

Our data suggests that upregulated glutathione may play both beneficial and pathogenic roles in PWB lesions. Glutathione is the most abundant non-protein thiol in the cell [30,31,32]. The synthesis of glutathione is regulated by many metabolic pathways, including the glutamine, cysteine, glycine, glutamate, pentose, etc., pathways [33,34,35]. Many of these pathways are dysregulated in PWB iPSCs from the KEGG data. In addition, growth factors, commons stressors, and cellular metabolic reprogramming also regulate intracellular glutathione levels [31,33,36,37,38]. Glutathione plays major beneficial roles in maintaining cellular redox homeostasis by acting as a free radical scavenger and a detoxifying agent; it contributes to multiple cellular processes including proliferation, cell division, and differentiation [31,32]. Alternatively, dysregulated glutathione metabolism has been shown to have a pathogenic role in malignancy and many diseases [31,38,39]. Glutathione levels are increased in many types of tumor cells [40,41,42]. Excessive glutathione promotes tumor progression, correlates with increased metastasis, and increases chemo-therapeutic resistance [31,43,44,45,46,47]. The progressively dilated PWB vasculature is a low-flow vascular malformation, which results in persistently mild hypoxia in lesions. This is supported by the elevated levels of HIF-1α in this study. Mild hypoxic conditions lead to an increase in ROS levels in PWB lesions. This will facilitate the activation of proangiogenic pathways including MAPK, JNK, PKC, and PI3K/AKT. In addition, increased glutathione will scavenge excessive oxidation and maintain an intricate antioxidant status for PWB vascular cells. A high level of glutathione can directly scavenge laser-induced free radicals such as reactive oxygen species (ROS) and nitric oxide (NO) [48,49], thus attenuating treatment efficacy. Our data suggests that the unexplored hypoxia/ROS/glutathione signaling axis may act as an important factor in the progressive pathological development and laser-resistant phenotypes of PWB. This will be the focus of our next studies. 

There are several limitations to this study. First, it is essential to determine metabolomes from PWB iPSC-derived ECs and skin lesions. Though the upregulated HIF-1α and enzymes regulating glutathione metabolism indicate an elevation of glutathione in PWB lesions, it is unknown whether PWB patients have perturbative glutathione and sphingolipid metabolisms and increased ROS levels in their skin lesions. Furthermore, the expression patterns of the enzymes responsible for sphingosine metabolism are yet to be examined. These important questions will be addressed in our future work. Second, the inter-individual and inter-sample variances were not sufficiently addressed in this study due to the small sample size. This might result in a lack of power for many metabolites with high variances among samples. A follow-up study with a larger sample size including both biological and experimental replications is planned as part of future works. Third, non-targeted metabolomics covers a wide range of metabolites, but the absolute quantification of metabolites is lacking. A follow-up targeted metabolomic study for glutathione and sphingolipid metabolism is needed to validate the current data. Fourth, the exact link between perturbed metabolites and pathology in lesions is yet to be determined, which will be our next focus. Fifth, we only focused on sphingosine and glutathione in this study; the study of other DMs such as pentose and glucuronate interconversions; amino sugars and nucleotide sugars; alanine, aspartate, and glutamate; and arginine, purine, D-glutamine, and D-glutamate were not performed. This prioritization was based on the following postulations: (1) decreased sphingosine could regulate the activation of the MAPK and PI3K pathways in the PWB lesions and (2) increased glutathione could result in the effective detoxification of ROS, thus attenuating the treatment efficacy of PDL in patients. We then further examined the glutathione pathways in PWB lesions so that such information could directly benefit patients undergoing regular PDL treatment. However, the potential functions of other DMs in PWB pathology should not be underestimated or neglected, which are worthy of future investigation. Sixth, the PWB iPSC lines generated in this study were from two patients; however, these samples cannot represent the whole patient population. Laser-resistant phenotypes are heterogeneous among PWB patients as well as in different subtypes of the vasculature within an individual’s PWB lesion. Therefore, any generalization of these conclusions to a broader patient population needs to be done with caution. Additionally, validation studies with larger sample sizes are required prior to the application of these results to patient care. 

In summary, we have found that sphingosine and glutathione are perturbed in PWB iPSCs. PWB vasculatures are under persistent and mild hypoxia. Our current data demonstrate that there are dysregulated cellular redox homeostasis and sphingolipid-mediated signalosomes in the PWB vasculature, which may facilitate cell survival and pathological progression. Furthermore, elevated glutathione levels may contribute to laser-resistant phenotypes in the PWB vasculature.

## Figures and Tables

**Table 1:** DMs in PWB iPSCs as compared to control iPSCs.

ESI − Mode					ESI + Mode				
Metabolite	HMDB_ID	VIP	Log_2_(FC)	*p* Value	Metabolite	HMDB_ID	VIP	Log_2_(FC)	*p* Value
Glutamine	HMDB000641	2.914	−1.742	0.013	Indoline	HMDB253472	1.891	−0.876	0.022
15-Hydro-peroxy-EPE	HMDB062295	3.286	−1.937	0.002	Asymmetric dimethylarginine	HMDB001539	1.921	−0.916	0.015
					Phenylalanylglycine	HMDB028995	2.554	−1.419	0.005
					N-Heptanoylglycine	HMDB013010	2.446	−1.648	0.031
					15-Hydroperoxy-EPE	HMDB062295	2.675	−1.561	0.012
					1,5,8-Heptadecatriene	HMDB041082	1.868	−0.770	0.001
					Histidylleucine	HMDB028889	2.820	−1.660	<0.001
					Sphingosine	HMDB000252	1.767	−0.736	0.003
Thyronine	HMDB000667	1.733	0.635	0.013	2-Heptadecylfuran	HMDB033608	1.562	0.802	0.045
Glutathione	HMDB000125	1.867	0.710	0.009	Glutathione	HMDB000125	1.549	0.677	0.046
Islatravir	HMDB253596	1.870	0.691	0.010	Thromboxane	HMDB003208	1.774	0.969	0.037
CDP	HMDB001546	1.953	0.691	0.002	11-Eicosen-1-ol	HMDB034933	1.817	0.822	0.044
Uridine diphosphate glucose	HMDB000286	1.558	0.5164	0.018	N-(14-Methylhexadecanoyl)pyrrolidine	HMDB034373	1.803	1.030	0.043
Zeatin riboside triphosphate	HMDB304509	1.645	0.594	0.021	Pipericine	HMDB031678	1.686	0.908	0.049
					4,6-Heneicosanedione	HMDB035571	2.106	1.538	0.031
					N-Palmitoyl Leucine	HMDB241928	1.537	0.629	0.048

Note: HMDB: the Human Metabolome Database; VIP: variable projection importance score (VIP > 1.5) of the metabolite in Partial Least Squares Discriminant Analysis model; Log2(FC): the log2 of fold change of the metabolites in PWB iPSCs vs. control iPSCs. If the value is greater than 0, the content of the metabolite is increased in the PWB iPSCs. *p* value: a statistic result from *t*-test comparing PWB iPSCs with the control iPSCs.

## Data Availability

Additional data are available in the supplementary data file in this study or/and can be directly quested to the authors.

## Supplementary Materials

The following supporting information can be downloaded at: https://www.mdpi.com/article/10.3390/metabo13090983/s1, Figure S1A: TIC of metabolome analysis by LC-MS in ESI – mode; QC, *n* = 5; Figure S1B: TIC for PWB_3921_9 in ESI – mode; Figure S1C: TIC for PWB_3921_9d in ESI – mode; Figure S1D: TIC for PWB_3921_16 in ESI – mode; Figure S1E: TIC for PWB_4221_3 in ESI – mode; Figure S1F: TIC for PWB_4221_3d in ESI – mode; Figure S1G: TIC for PWB_4221_6 in ESI – mode; Figure S1H: TIC for PWB_52521_8 in ESI – mode; Figure S1I: TIC for PWB_52521_8d in ESI – mode; Figure S1J: TIC for PWB_52521_9 in ESI – mode; Figure S2A: TIC of metabolome analysis by LC-MS in ESI + mode; QC, *n* = 5; Figure S2B: TIC for PWB_3921_9 in ESI + mode; Figure S2C: TIC for PWB_3921_9d in ESI + mode; Figure S2D: TIC for PWB_3921_16 in ESI + mode; Figure S2E: TIC for PWB_4221_3 in ESI + mode; Figure S2F: TIC for PWB_4221_3d in ESI + mode; Figure S2G: TIC for PWB_4221_6 in ESI + mode; Figure S2H: TIC for PWB_52821_8 in ESI + mode; Figure S2I: TIC for PWB_52821_8d in ESI + mode; Figure S2J: TIC for PWB_52821_9 in ESI + mode; Figure S3A: The scores scatter plot of PCA model in ESI – mode; Figure S3B: The scores scatter plot of PCA model in ESI + mode; Figure S4A: The scores scatter plot of PLS-DA model in ESI – mode; Figure S4B: The scores scatter plot of PLS-DA model in ESI + mode; Figure S5A: The scores scatter plot of OPLS-DA model in ESI – mode; Figure S5B: The scores scatter plot of OPLS-DA model in ESI + mode; Figure S6A: The loading plots of PLSDA model in ESI – mode. Red box: metabolites with VIP > 1.5; Figure S6B: The loading plots of PLSDA model in ESI + mode. Red box: metabolites with VIP > 1.5; Figure S7: Original images for IHC. Dashed blue boxed areas were used in the Figure 5 in each panel; Table S1: The profile of the fragments identified in Figure 3.
