# Supplementary material for: Perturbations of Glutathione and Sphingosine Metabolites in Port Wine Birthmark Patient-Derived Induced Pluripotent Stem Cells
Source: Metabolites. 2023 Aug 31;13(9):983. doi: 10.3390/metabo13090983 (PMC10537749; doi:10.3390/metabo13090983)

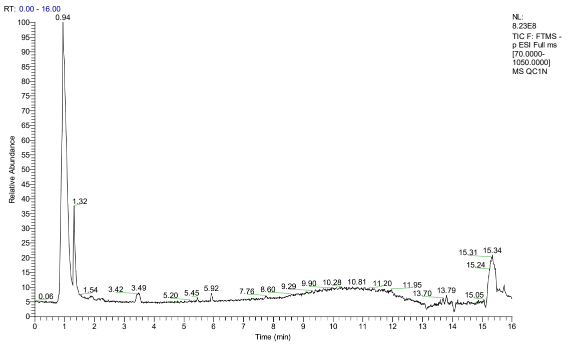

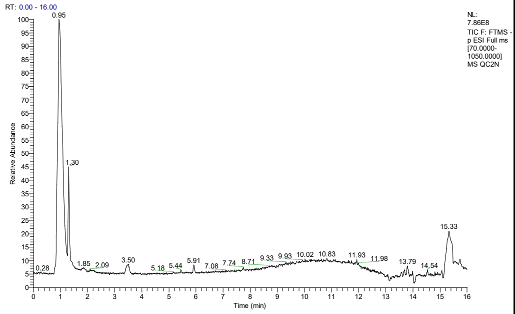

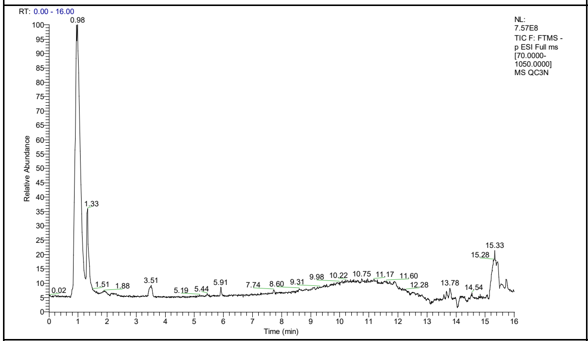

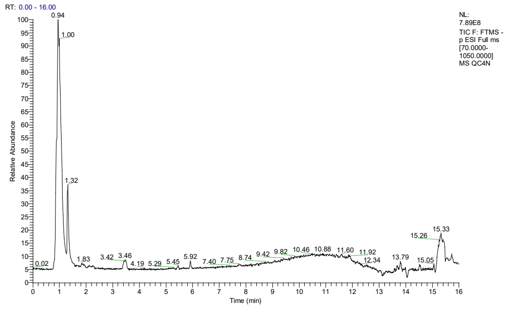

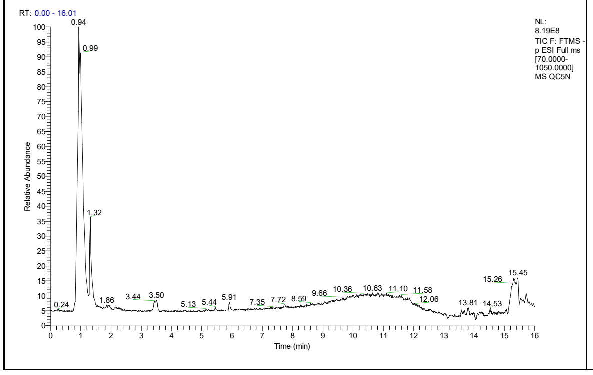

Suppl. Fig 1A, TIC of metabolome analysis by LC-MS in ESI – mode; QC, n=5

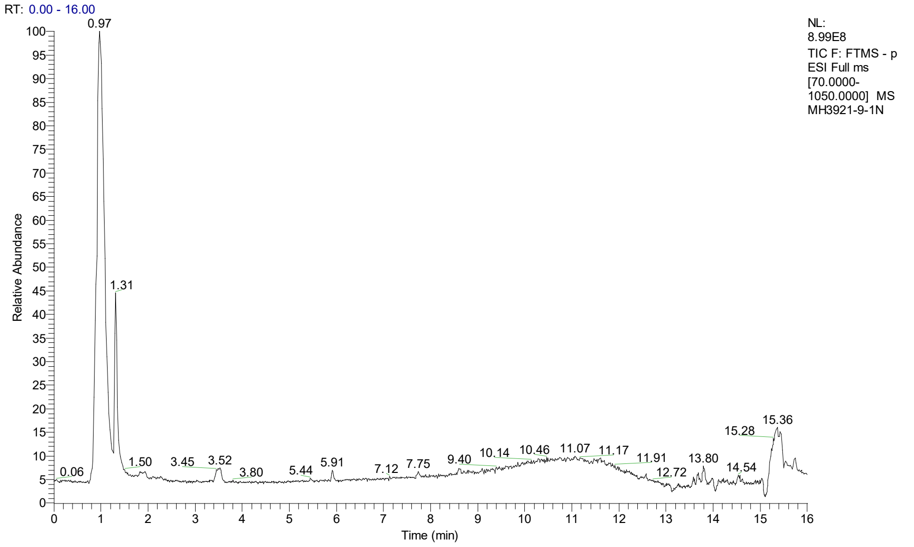

Suppl. Fig 1 B, TIC for PWB_3921_9 in ESI - mode.

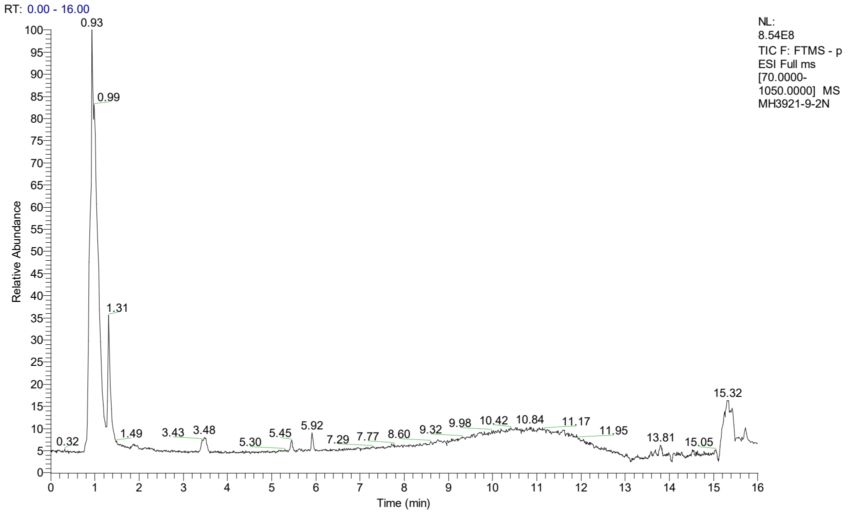

Suppl. Fig 1 C, TIC for PWB_3921_9d in ESI - mode.

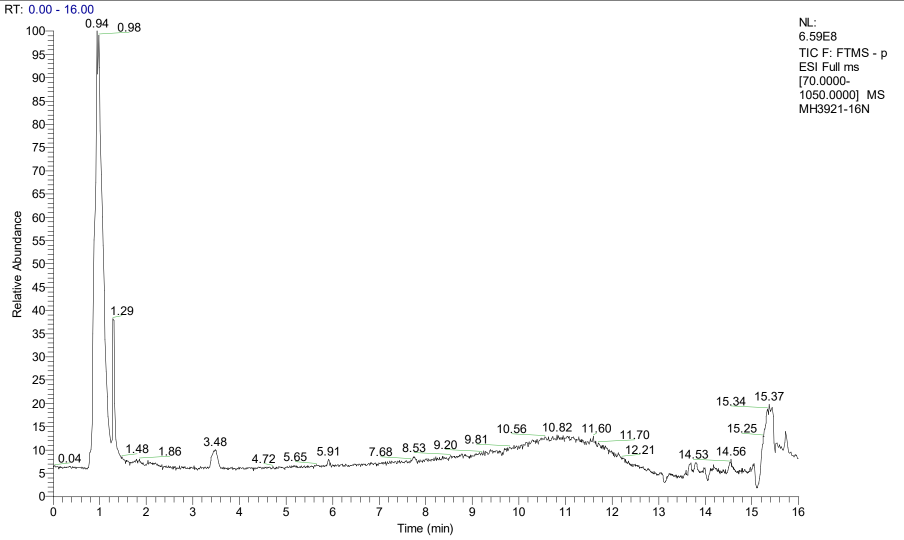

Suppl. Fig 1 D, TIC for PWB_3921_16 in ESI - mode.

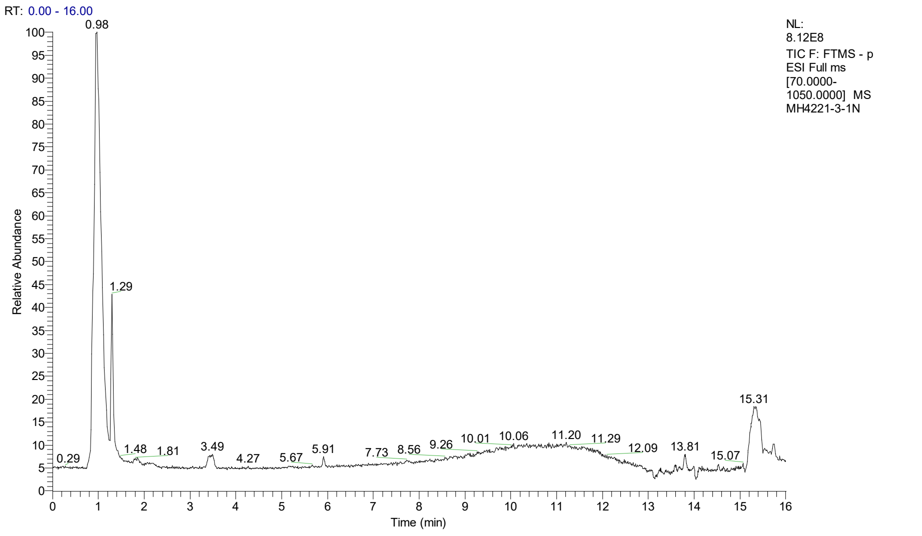

Suppl. Fig 1 E, TIC for PWB_4221_3 in ESI - mode.

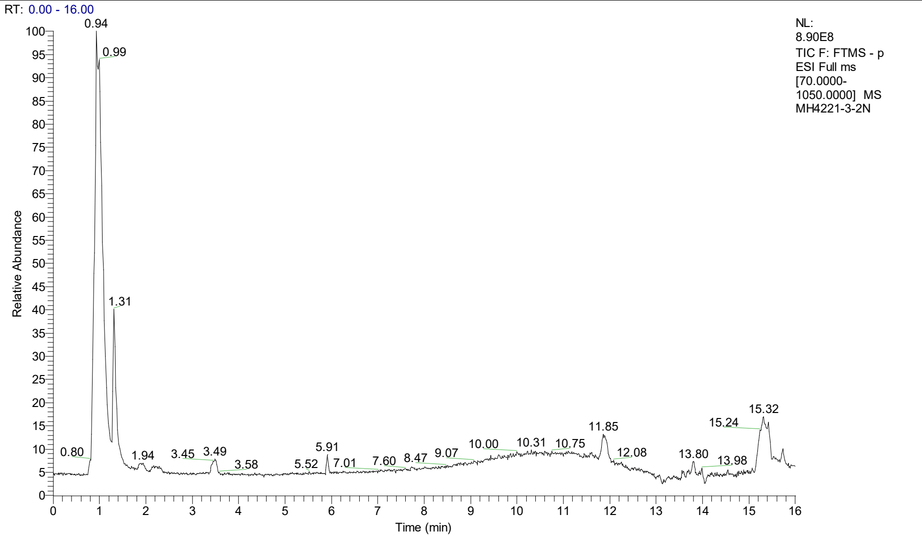

Suppl. Fig 1 F, TIC for PWB_4221_3d in ESI - mode.

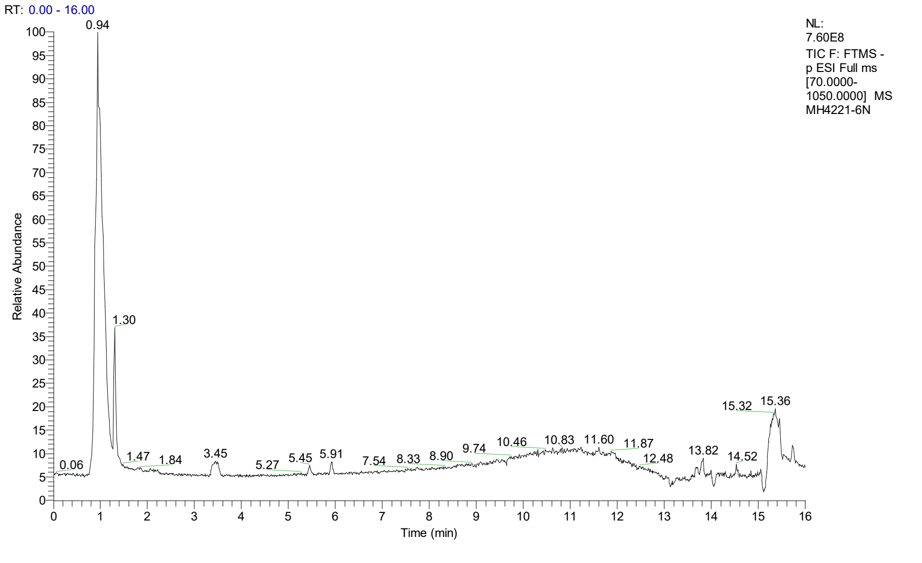

Suppl. Fig 1 G, TIC for PWB_4221_6 in ESI - mode.

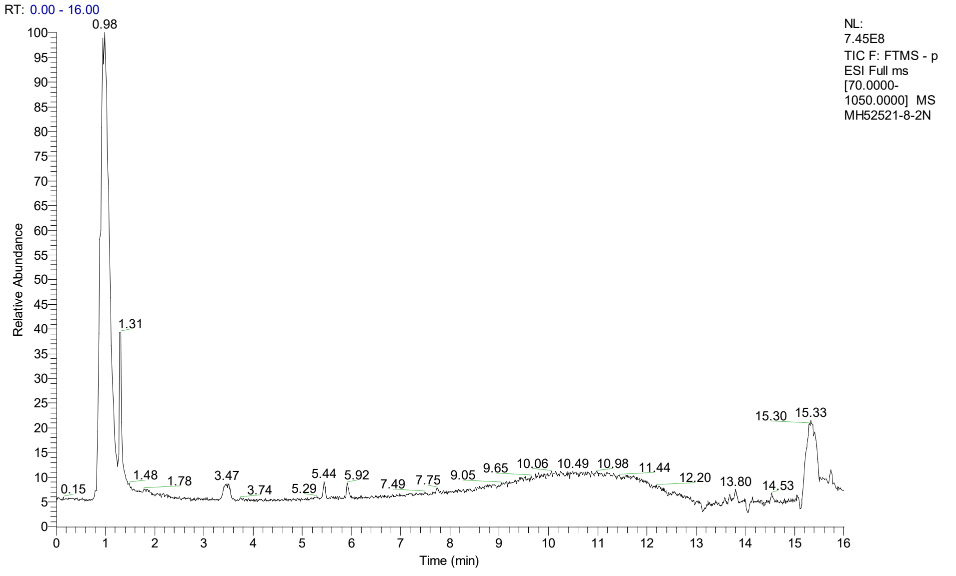

Suppl. Fig 1 H, TIC for PWB_52521_8 in ESI - mode.

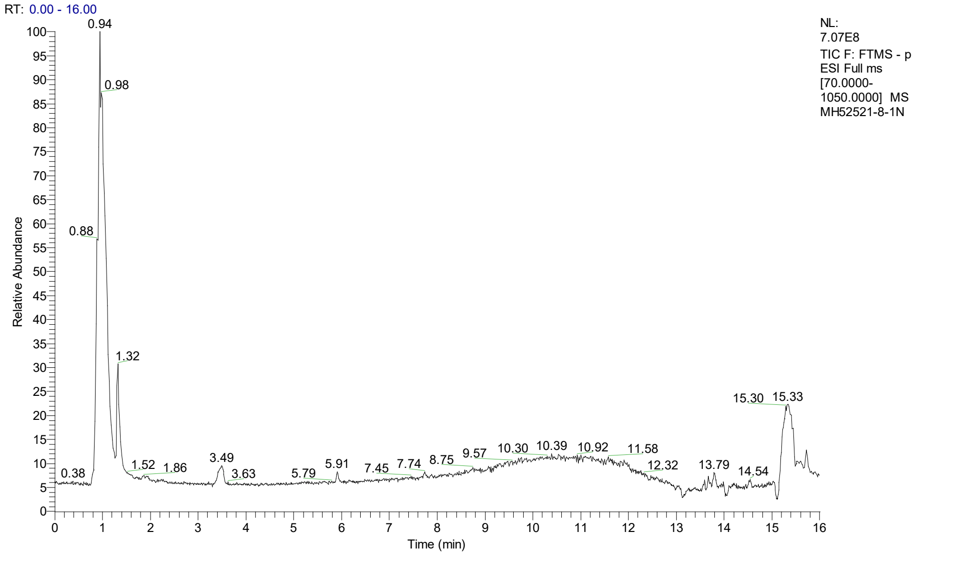

Suppl. Fig 1 I, TIC for PWB_52521_8d in ESI - mode.

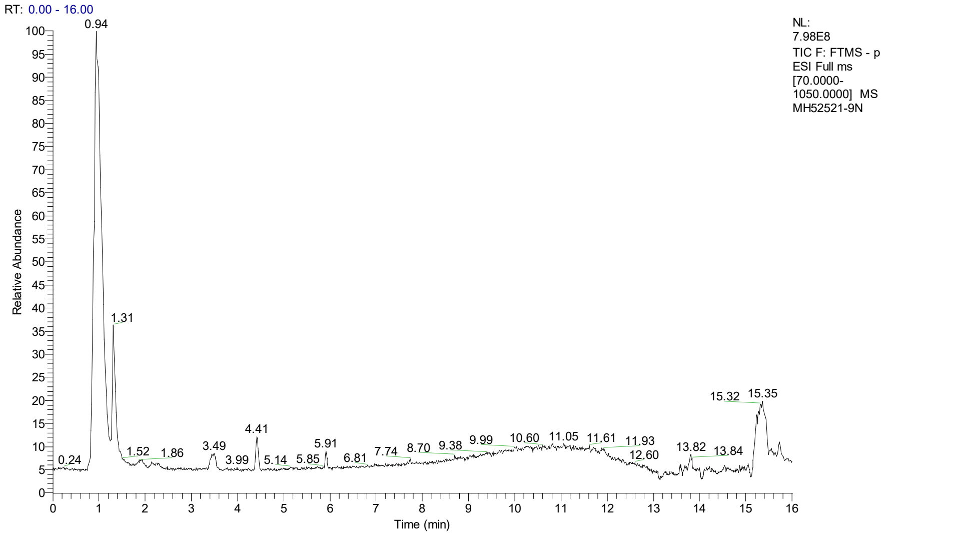

Suppl. Fig 1 J, TIC for PWB_52521_9 in ESI - mode.

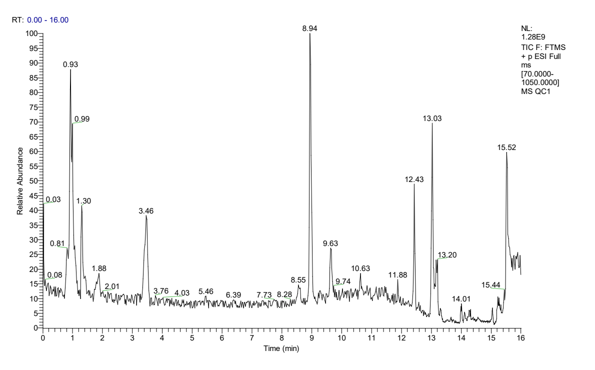

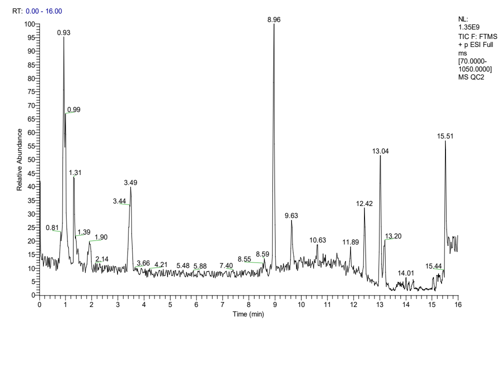

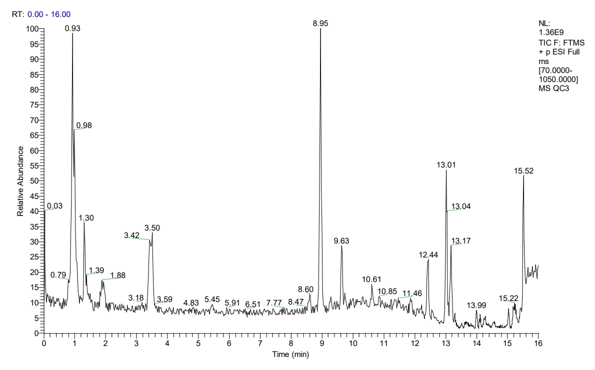

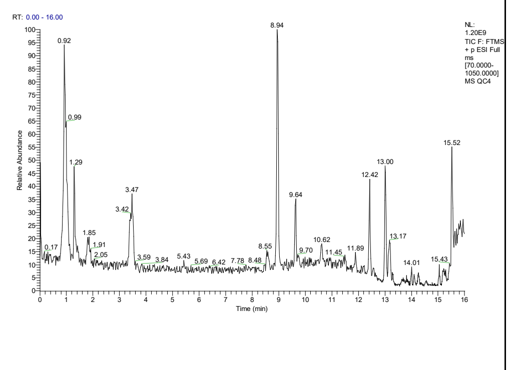

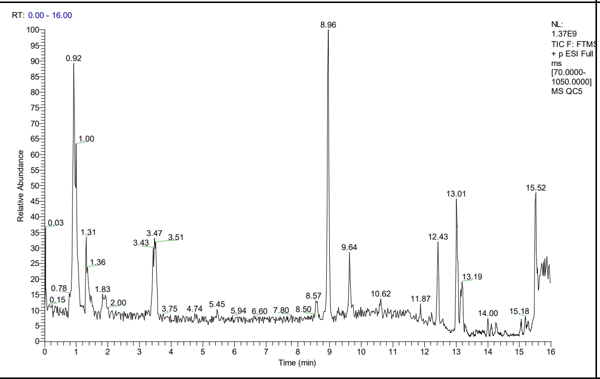

Suppl. Fig 2A, TIC of metabolome analysis by LC-MS in ESI + mode; QC, n=5

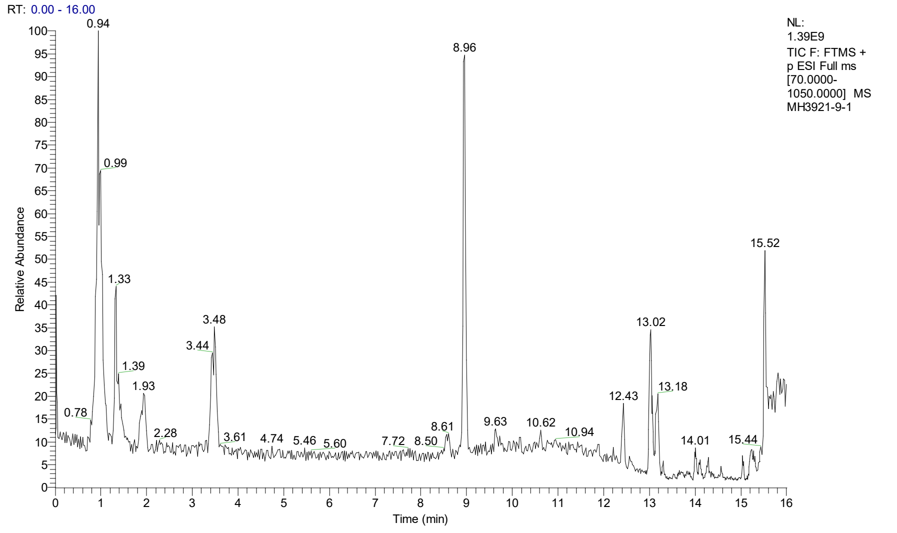

Suppl. Fig 2 B, TIC for PWB_3921_9 in ESI + mode.

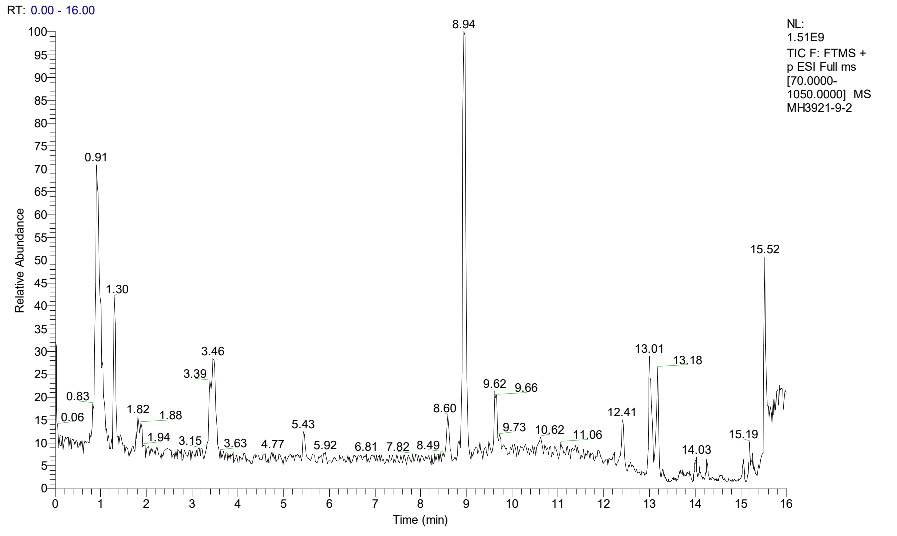

Suppl. Fig 2 C, TIC for PWB_3921_9d in ESI + mode.

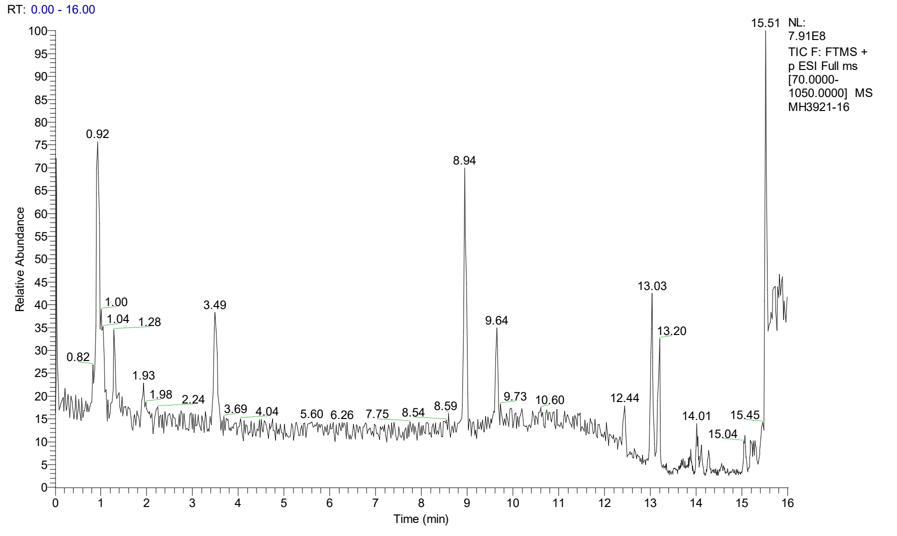

Suppl. Fig 2 D, TIC for PWB_3921_16 in ESI + mode.

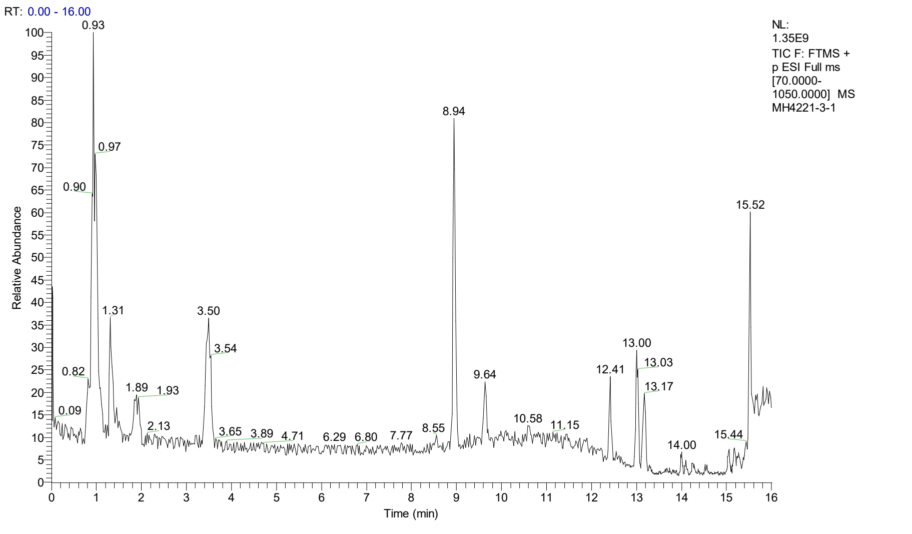

Suppl. Fig 2 E, TIC for PWB_4221_3 in ESI + mode.

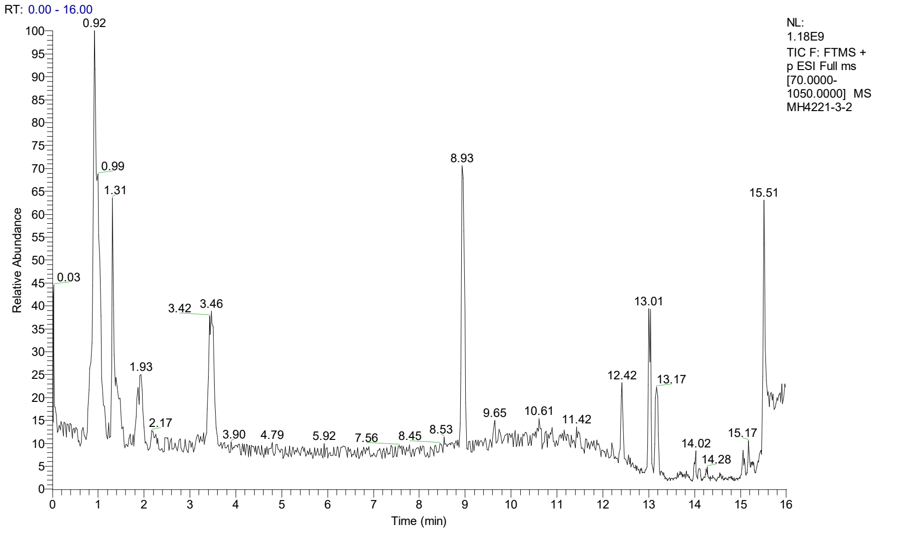

Suppl. Fig 2 F, TIC for PWB_4221_3d in ESI + mode.

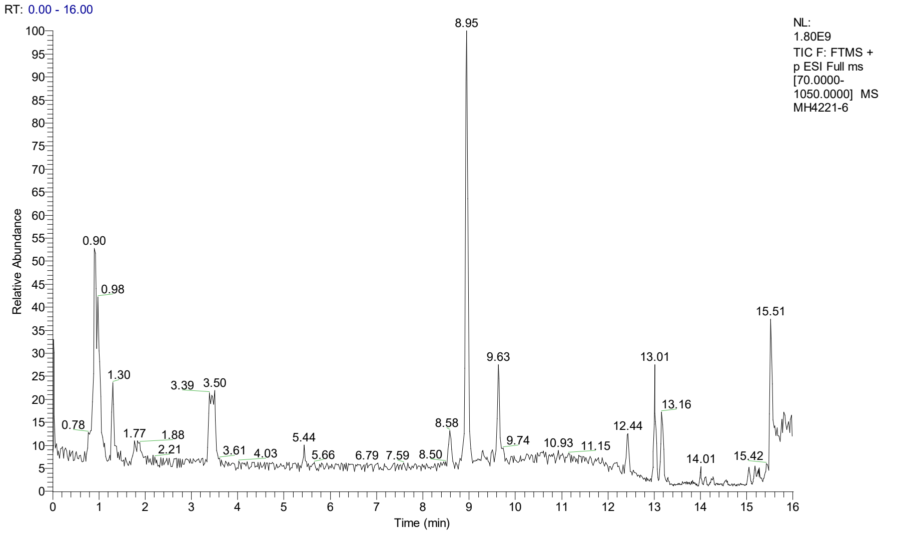

Suppl. Fig 2 G, TIC for PWB_4221_6 in ESI + mode.

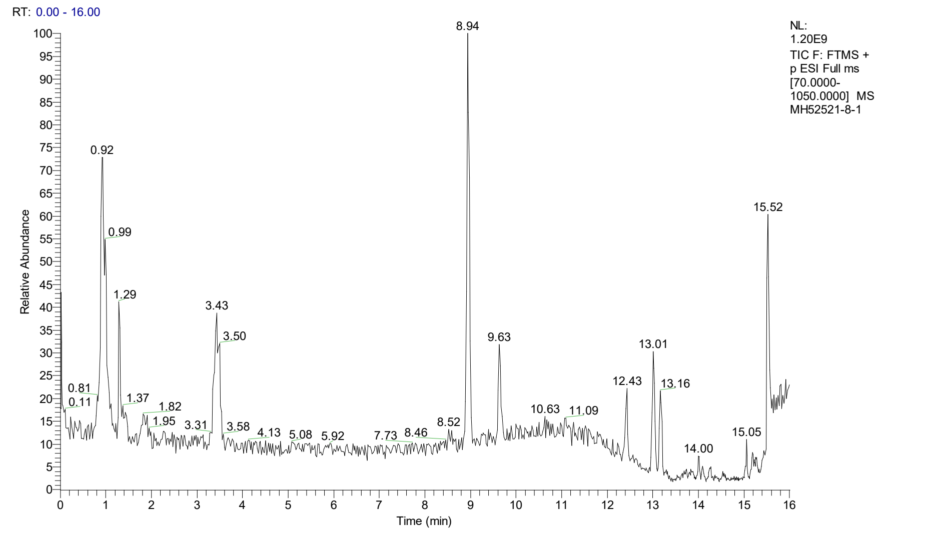

Suppl. Fig 2 H, TIC for PWB_52821_8 in ESI + mode.

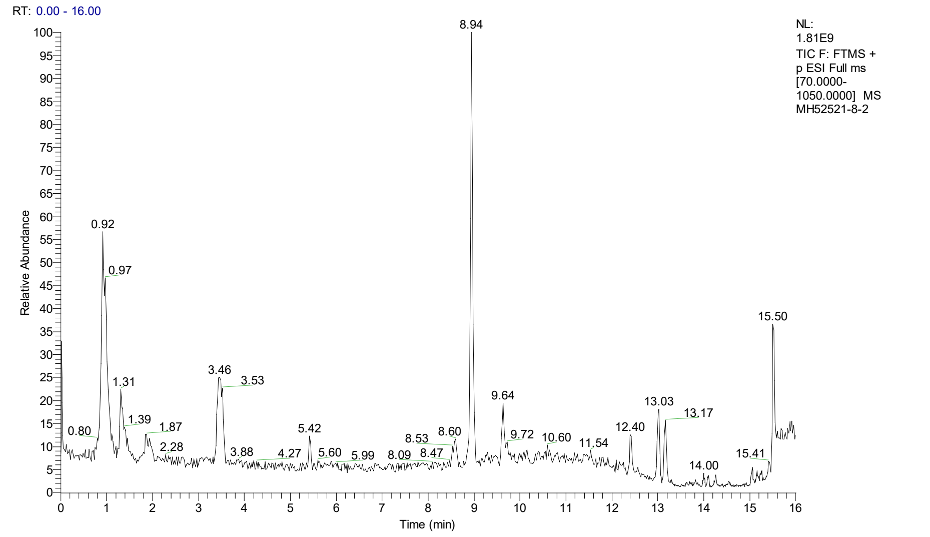

Suppl. Fig 2 I, TIC for PWB_52821_8d in ESI + mode.

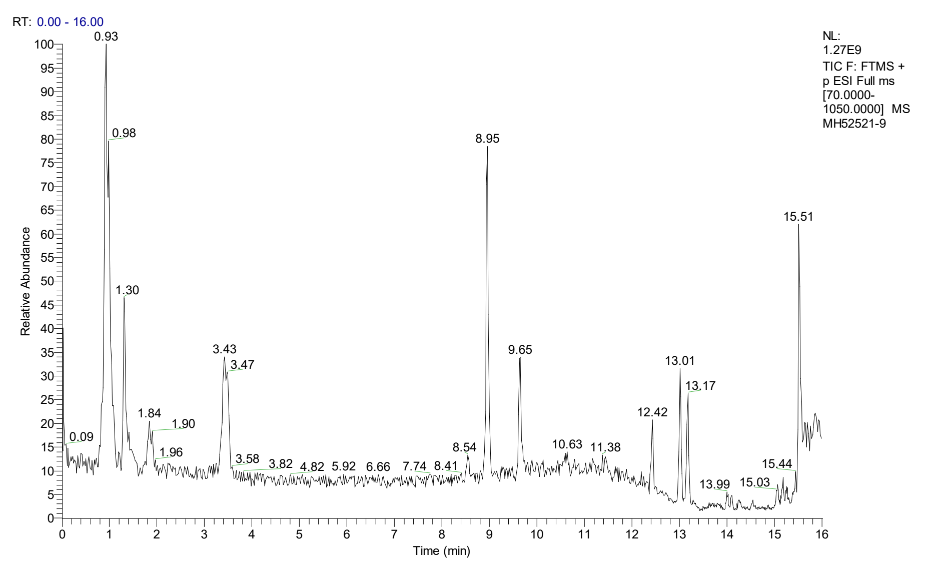

Suppl. Fig 2 J, TIC for PWB_52821_9 in ESI + mode.

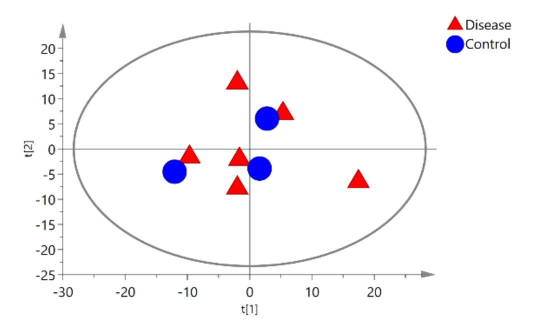

Suppl Fig 3A. The scores scatter plot of PCA model in ESI – mode.

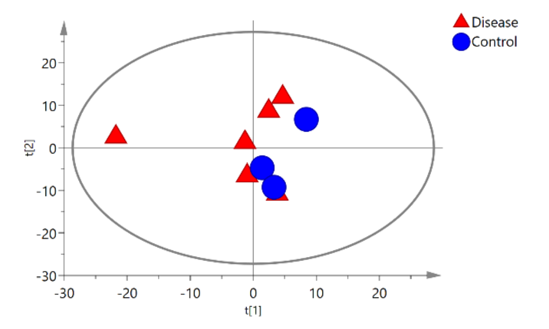

Suppl Fig 3B. The scores scatter plot of PCA model in ESI + mode.

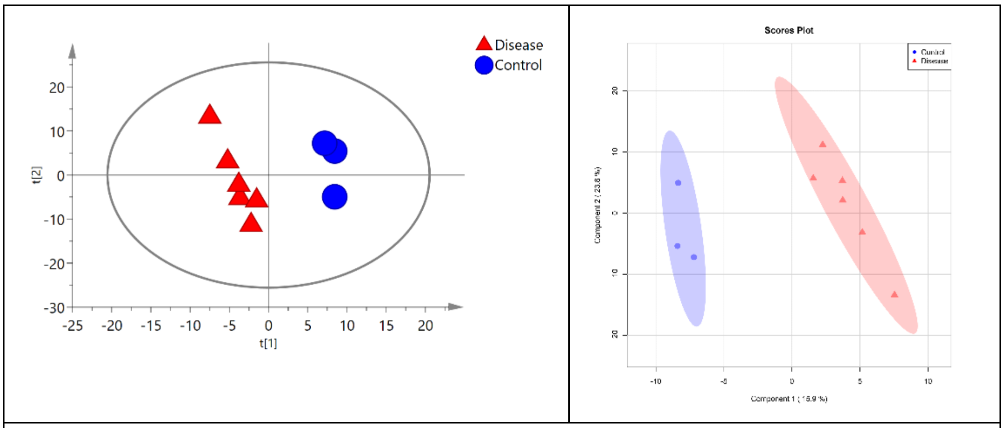

Suppl Fig 4A. The scores scatter plot of PLS-DA model in ESI – mode.

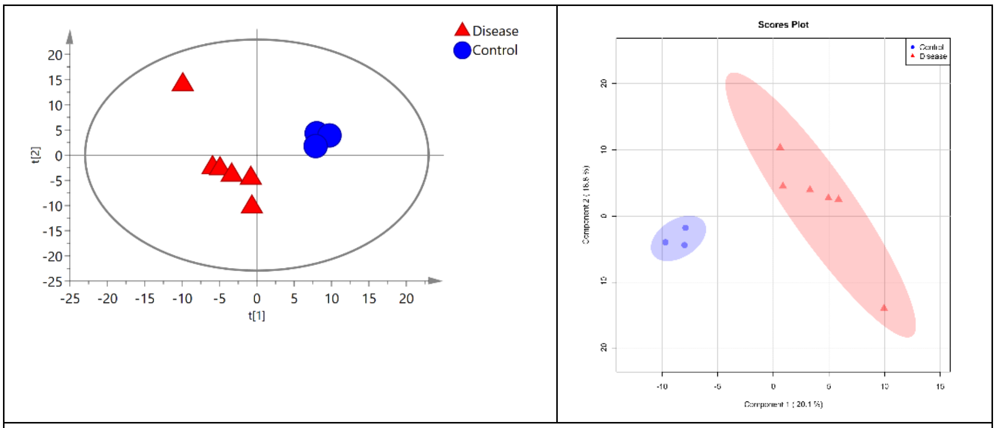

Suppl Fig 4B. The scores scatter plot of PLS-DA model in ESI + mode.

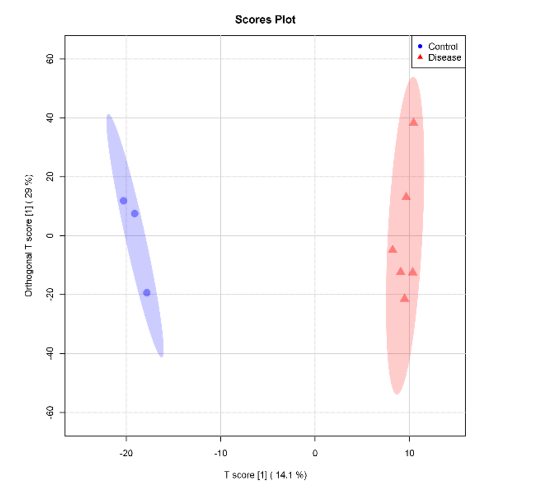

Suppl Fig 5A. The scores scatter plot of OPLS-DA model in ESI – mode.

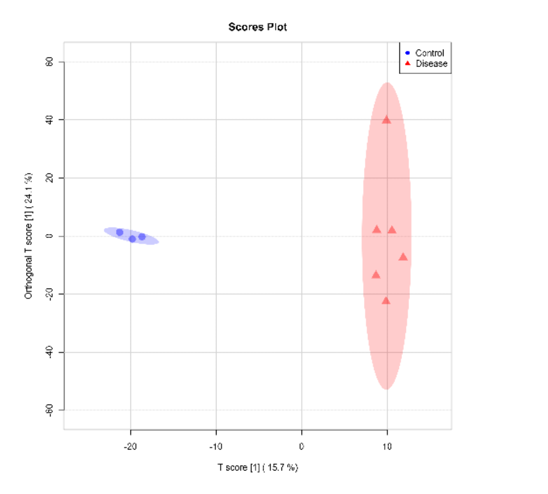

Suppl Fig 5B. The scores scatter plot of OPLS-DA model in ESI + mode.

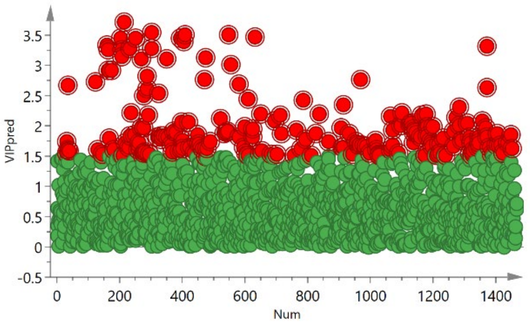	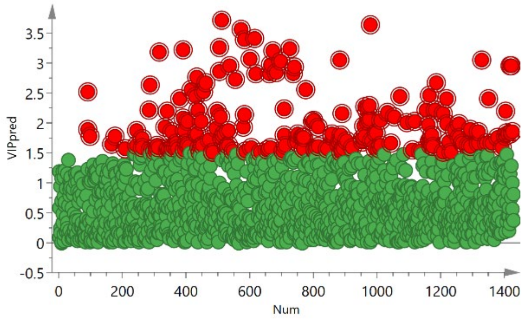
**Suppl Fig 6A. The loading plots of PLS-DA model in ESI – mode. Red box: metabolites with VIP > 1.5.**	**Suppl Fig 6B. The loading plots of PLS-DA model in ESI + mode. Red box: metabolites with VIP > 1.5.**

Supplementary Table 1: The profile of the fragments identified in Fig 3

Metabolites	Fragment formula	Fragment m/z	Peak m/z	Error (ppm)	Charge	Neutral charge
Sphingolipid_ESI+	C4H9-e	57.0699	57.0699	0.99	1	
	C2H6NO-e	60.0444	60.0445	1.81	1	
	C5H11-e	71.0856	71.0850	-7.34	1	
	C4H7NO+H	86.0601	86.0598	-3.49	1	
	C6H10O-e	97.0648	97.0645	-2.37	1	-H
	C6H10NO+H	114.0914	114.0914	-0.13	1	+H
	C17H32N+H	252.2686	252.2702	6.45	1	+H
	C18H34N-e	264.2686	264.2683	-1.03	1	
	C17H34NO+H	270.2792	270.2769	-8.25	1	+H
	C18H36NO-e	282.2792	282.2784	-2.51	1	
	C18H37NO2+H	300.2897	300.2887	-3.37	1	
Glutathione_ESI+	C2H4NO2+H	76.0393	76.0389	-5.42	1	+H
	C4H6NO-e	84.0444	84.0442	-1.75	1	
	C3H2NO2+H	85.0159	85.0159	0.72	1	
	C5H8NO3-e	130.0499	130.0497	-0.83	1	
	C5H6NO2S-e	144.0114	144.0114	0.11	1	
	C5H8NO3S-e	162.0220	162.0216	-1.94	1	
	C5H9N2O3S+H	179.0485	179.0477	-4.09	1	+H
	C8H11N2O3S-e	215.0485	215.0487	1.18	1	
	C8H13N2O4S-e	233.0591	233.0577	-5.89	1	
	C9H13N2O4S-e	245.0591	245.0582	-3.67	1	
	C10H15N2O6S-e	291.0646	291.0646	0.92	1	
	C10H17N3O6S+H	308.0911	308.0895	-5.09	1	
Glutathione_ESI-	C2H4NO2-H	72.0091	72.0090	-1.47	1	
	C2H4NO2+e	74.0247	74.0249	2.70	1	
	C3H6NO2-H	86.0247	86.0248	0.84	1	-H
	C5H6NO2+e	112.0404	112.0406	1.32	1	
	C5H6NO3+e	128.0353	128.0354	0.64	1	
	C5H9N2O3-H	143.0462	143.0464	0.93	1	-H
	C5H8NO3S-H	160.0074	160.0074	0.14	1	-H
	C5H9N2O3S+e	177.0339	177.0337	-1.63	1	
	C7H10N2O4-H	185.0568	185.0565	-1.57	1	
	C9H12N3O5+e	242.0782	242.0788	2.20	1	
	C10H14N3O5-H	254.0782	254.0777	-2.19	1	-H
	C10H16N3O6-H	272.0888	272.0884	-1.63	1	-H
	C10H15N3O5S-H	288.0659	288.0656	-0.97	1	
	C10H17N3O6S-H	306.0765	306.0764	-0.55	1	

Suppl Fig 7 Original images for IHC. Dashed blue boxed areas were used in the Fig 5 in each panel.

A. HIF-1α

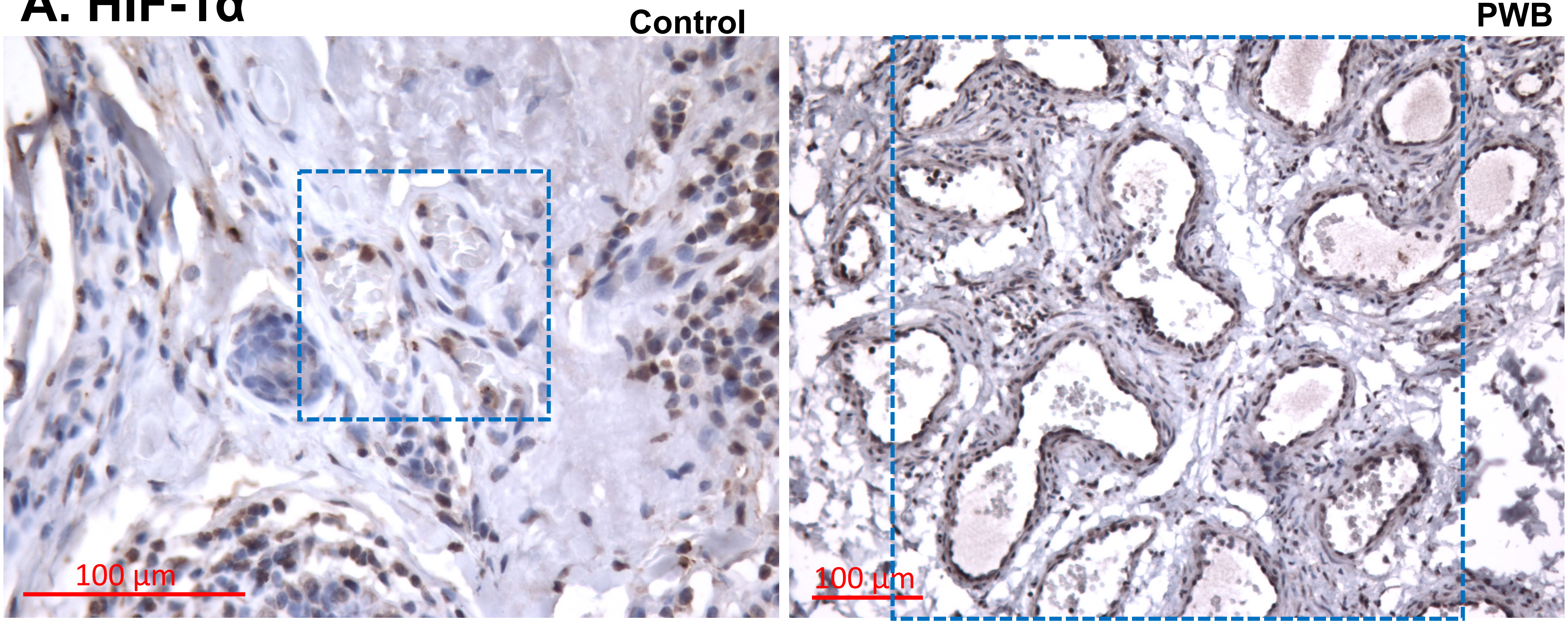

B. GCLM

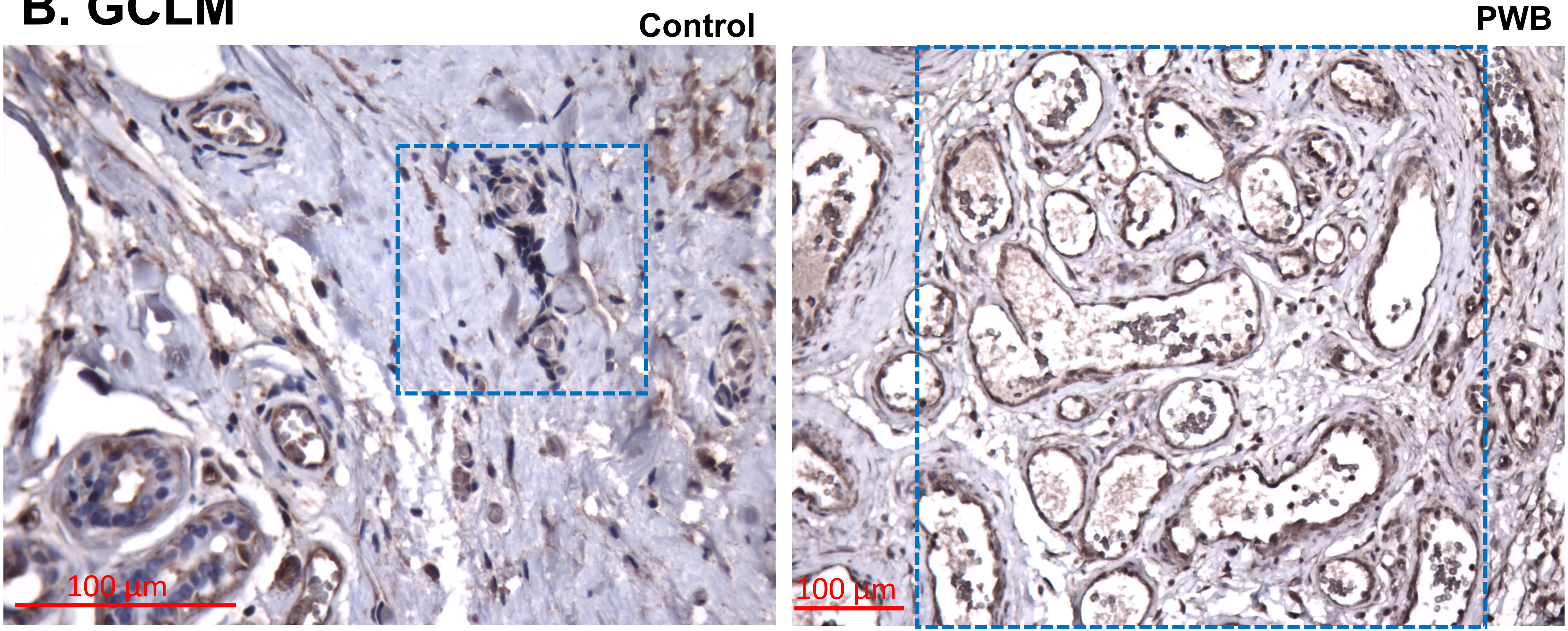

C. GGT7

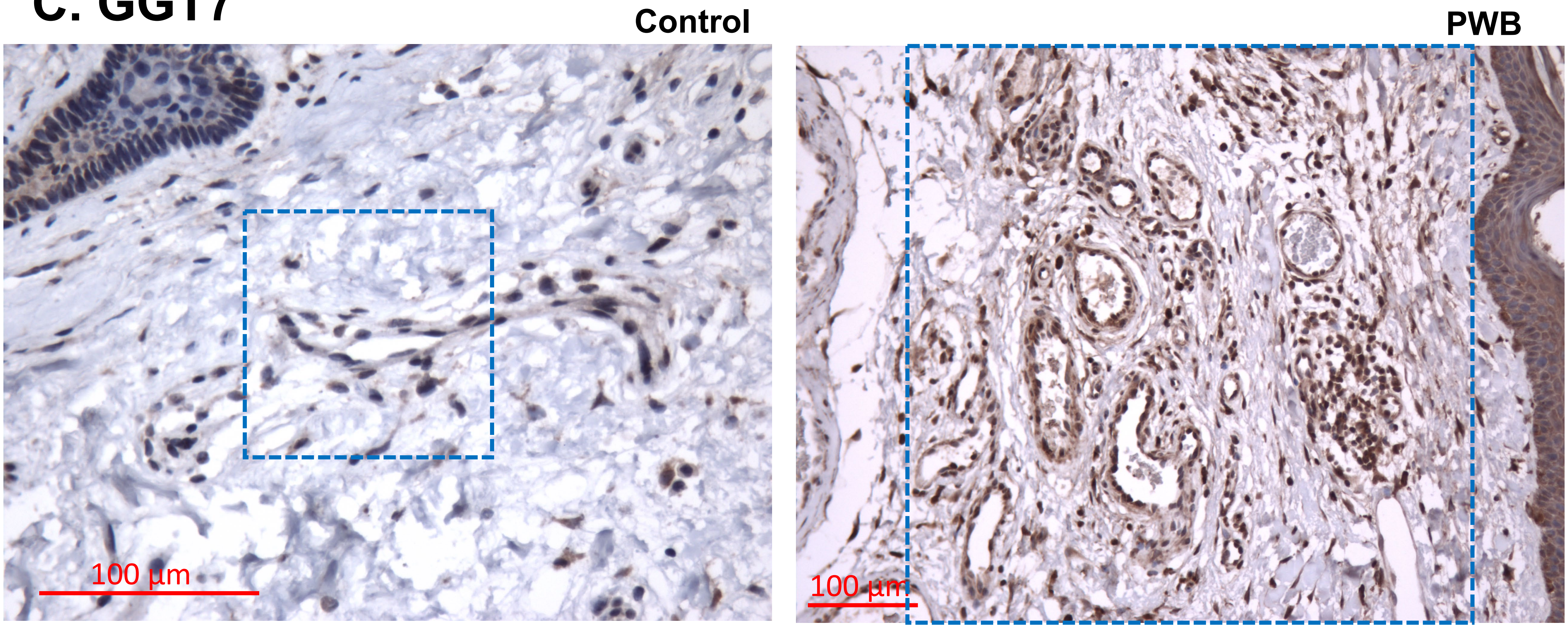

D. GSTP1

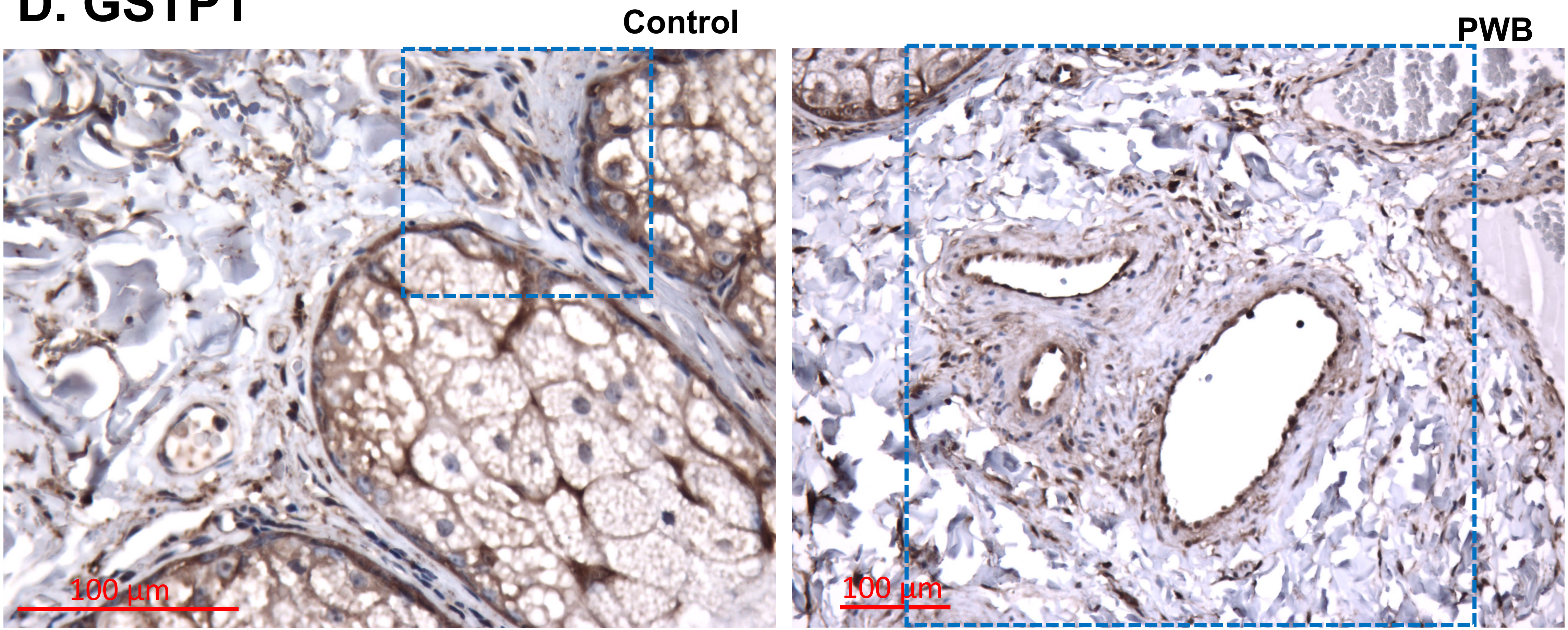

Supplement: Supplementary file 1 [file metabolites-13-00983-s001.zip › metabolites-2539715-supplementary.pdf]
